# Life history strategies of *Cotylurus* spp. Szidat, 1928 (Trematoda, Strigeidae) in the molecular era – Evolutionary consequences and implications for taxonomy

**DOI:** 10.1016/j.ijppaw.2022.06.002

**Published:** 2022-06-11

**Authors:** Ewa Pyrka, Gerard Kanarek, Julia Gabrysiak, Witold Jeżewski, Anna Cichy, Anna Stanicka, Elżbieta Żbikowska, Grzegorz Zaleśny, Joanna Hildebrand

**Affiliations:** a Department of Parasitology, Faculty of Biological Sciences, University of Wrocław, Przybyszewskiego 63, 51-148, Wrocław, Poland; b Ornithological Station, Museum and Institute of Zoology, Polish Academy of Sciences, Nadwiślańska 108, 80-680, Gdańsk, Poland; c Witold Stefański Institute of Parasitology, Polish Academy of Sciences, Twarda 51/55, 00-818, Warszawa, Poland; d Department of Invertebrate Zoology and Parasitology, Faculty of Biology and Environment Protection, Nicolaus Copernicus University in Toruń, Lwowska 1, 87-100, Toruń, Poland; e Department of Systematic and Ecology of Invertebrates, Institute of Environmental Biology, Wrocław University of Environmental and Life Sciences, Kożuchowska 5b, 51-631, Wrocław, Poland

**Keywords:** Strigeidae, Snails, Metacercariae, Tetracotyle, *Cotylurus*

## Abstract

Species of *Cotylurus* Szidat, 1928 (Diplostomoidea: Strigeidae) are highly specialized digeneans that parasitize the gastrointestinal tract and bursa of Fabricius of water and wading birds. They have a three-host life cycle; the role of first intermediate host is played by pulmonate snails, while a wide range of water snails (both pulmonate and prosobranch) and leeches are reported as second intermediate hosts.

Unfortunately, species richness, molecular diversity and phylogeny of metacercariae of *Cotylurus* spp. (tetracotyle) occurring in snails remain poorly understood. Thus, we have performed the parasitological and taxonomical examination of tetracotyles form freshwater snails from Poland, supplemented with adult Strigeidae specimens sampled from water birds. In this study we report our use of recently obtained sequences of two molecular markers (28S nuclear large ribosomal subunit gene (28S rDNA) and the cytochrome *c* oxidase subunit 1 (CO1) fragment), supplemented by results of a method of species delimitation (GMYC) and haplotype analysis to analyse some aspects of the ecology, taxonomy, and phylogeny of members of the genus *Cotylurus.*

The provided phylogenetic reconstructions discovered unexpectedly high molecular diversity within *Cotylurus* occurring in snails, with clearly expressed evidence of cryptic diversity and the existence of several novel-species lineages. The obtained results revealed the polyphyletic character of *C. syrius* Dubois, 1934 (with three separate molecular species-level lineages) and *C. cornutus* (Rudolphi, 1809) Szidat, 1928 (with four separate molecular species-level lineages). Moreover, we demonstrated the existence of two divergent phylogenetical and ecological lineages within *Cotylurus* (one using leeches and other snails as second intermediate hosts), differing significantly in their life history strategies.

## Highlights

• Within *Cotylurus* existed two divergent phylogenetical and ecological lineages.
• The lineages within *Cotylurus* sp. differing significantly in their routes of transmission.
• Molecular analysis revealed high genetic diversity with evidence of cryptic species.

## Graphical abstract

**Figure:**
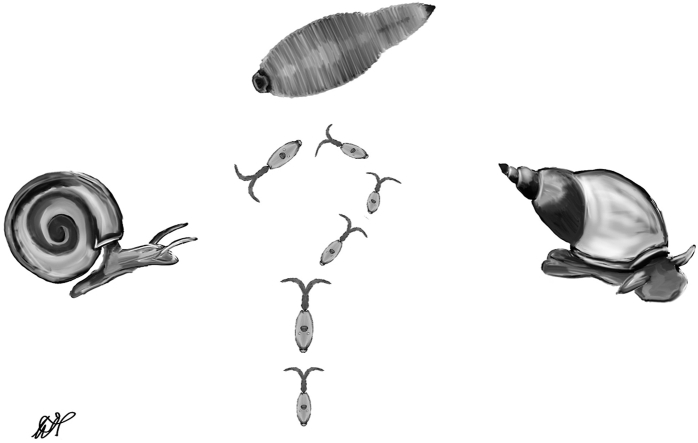

## 1 Introduction

The genus *Cotylurus* Szidat, 1928 (Diplostomoidea: Strigeidae) constitutes a relatively small group of highly specialized, widely distributed digeneans, specific parasites of the gastrointestinal tract and bursa of Fabricius of water and wading birds, usually Anseriformes, Charadriiformes and Gruiformes (Sudarikov, 1984; Sitko et al., 2006). *Cotylurus* possess a long and confusing history within Strigeidae, with systematic structure varying significantly among authors. The history of this genus has been described in detail in our previous work (Pyrka et al., 2021). *Cotylurus* is widely recognised as valid taxa (e.g., Niewiadomska, 2002) with independent status confirmed in recent molecular studies (Heneberg et al., 2018; Pyrka et al., 2021); however, the validity of particular species and real diversity within this taxon are still ambiguous. The adult forms of *Cotylurus* spp. are characterised by high levels of morphological variability, which has led some authors to taxonomic decisions of questionable validity (Dubois, 1968). But from the other hand, the existence of a polymorphic species with a wide range of final hosts and cosmopolitan distribution was also suggested (e.g., Zazornova, 2004, 2010). Indisputably, this situation raises several significant problems with the status of particular species, and moreover, precise and adequate delimiting of species within *Cotylurus*. Regarding these facts, Heneberg et al. (2018) have combined a morphological and molecular analysis of central European Strigeidae from avian definitive hosts to investigate the taxonomic structure of *Cotylurus*. Surprisingly, this research revealed a high level of molecular diversity within the morphologically well-established species (e.g., *C. syrius* Dubois, 1934), which strongly suggests its polyphyletic character. This has been confirmed in our recent study (Pyrka et al., 2021). Moreover, the mentioned studies affirm unclear taxonomic position type-species of *Cotylurus*, i.e., *C. cornutus* (Rudolphi, 1809) Szidat, 1928, which possibly indicates that this taxon *de facto* consists of a complex of species, as suggested earlier by Zazornova (2004, 2010). Recently, Locke et al. (2018), based on morphological features of adult trematode and molecular data, described a new species of *Cotylurus*, *C. marcogliesei* Locke et al., 2018, while other molecular analyses based on different larval stages (Gordy and Hanington, 2019; Nakao and Sasaki, 2021) reported the occurrence of several new molecular lineages within this genus. These data clearly proved that real species richness and the status of particular species within *Cotylurus* should be recognised as still far from being resolved and requiring further detailed morphological, molecular, and phylogenetic studies.

*Cotylurus* have a three-host life cycle; the role of first intermediate host is played by pulmonate snails (e.g., Sudarikov, 1959; Basch, 1969; Cichy et al., 2011; Sudarikov et al., 2002), while a wide range of water snails (both pulmonate and prosobranch) and leeches are reported as second intermediate hosts (e.g., Sudarikov et al., 2002; Urabe, 2001, 2003; Serbina, 2014; Pyrka et al., 2021). Metacercariae of the tetracotyle type are transmitted to the definitive host by ingestion of the second intermediate host (Sudarikov et al., 2002; Cribb et al., 2003; Blasco-Costa and Locke, 2017). In historical works, metacercariae of *Cotylurus*, usually classified as *Tetracotyle typica* de Fillippi, 1855, have been widely recorded from snails and leeches in Europe and generally identified as larval stages of *C. cornutus* (Szidat, 1929; Timon-David, 1943; Dobrowolski, 1958; for details see also Sudarikov, 1959 and references therein; Spelling and Young, 1986). Regarding these facts, *C. cornutus* was recognised as a taxon with a very broad specificity in relation to their host of invasive stages (e.g., Sudarikov, 1959; Dubois, 1968; Sudarikov et al., 2002). However, some authors (Sudarikov et al., 1962; Vojtek et al., 1967; Opravilová, 1969) suggest a different taxonomical position of tetracotyles collected from snails and leeches, although taxonomic consequences of morphological variability among tetracotyles of *Cotylurus* from different intermediate hosts have not been confirmed. The validity of these assumptions has been currently reported by Pyrka et al. (2021) who, based on comparative molecular analyses of tetracotyles sampled from leeches and adult trematodes collected from avian hosts, identified it as two species: *C. strigeoides* Dubois, 1958 and *C. syrius* Dubois, 1934. The data obtained in this research suggests a rather narrow host specificity within *Cotylurus* with respect to the second intermediate host and the existence of two separate ecological lineages: one utilising leeches and another utilising aquatic snails as second intermediate host. This situation raises several potentially serious evolutionary consequences for a wide range of host–parasite relationships, ecology, and taxonomy of this group of Digenea (Blasco-Costa and Poulin, 2017; Pyrka et al., 2021). From an evolutionary point of view, any modification of the complex life cycles of trematodes concerning switching/removal of an intermediate host can significantly change their success of transmission routes and raise key questions concerning some aspects of host-parasite relationships, especially their co-evolution (Poulin and Randhava, 2015; Blasco-Costa and Poulin, 2017; Kasl et al., 2018). Moreover, diverse life history strategies can be related to higher or lower energetic cost, duration of infection, separate dispersion strategies, etc., and can affect the success of transmission to intermediate and final hosts between particular species. The existence of these ecological lineages can shed new light on the structure of the *Cotylurus* and the phylogenetic relationships within. Additionally, their final resolve can give a completely new perspective on the biology and evolution of life cycles within Strigeidae.

Unlike the initially molecularly established taxonomic position of tetracotyles of *Cotylurus* from leeches (Pyrka et al., 2021), the issue of the identity and taxonomic position of tetracotyles occurring in snails remains still unresolved. Similar to tetracotyles from leeches, distinguishing a fully reliable species-level metacercariae of *Cotylurus* from snails is very difficult due to the high level of inter- and intra-specific homogeneity of morphological features that could enable their precise identification (Sudarikov, 1971, 1974; Zazornova, 1993, 2010). Therefore, little is known about the true diversity and host specificity within metacercariae of *Cotylurus*, which is directly related to important gaps in widely understood knowledge concerning the taxonomy, biology, and ecology of this genus. However, in recent decades, as an alternative to classical approaches, molecular techniques have been used efficiently in the identification and discrimination at all stages of trematodes, enabling full recognition of cryptic diversity (e.g., Detwiler et al., 2010; Georgieva et al., 2013a, 2013b; McNamara et al., 2014), evaluation of genetic variation (e.g., Brusentsov et al., 2013; Voronova and Chelomina, 2018; Tatonova et al., 2019; Schwantes et al., 2020), and inference of phylogenies (see e.g., Pérez-Ponce de León and Hernández-Mena, 2019 and references therein for details). The wide use of molecular techniques gives the unique possibility of a direct connection between the morphology of the adults and molecular records of the larval stages. Unfortunately, so far, only Soldánová et al. (2017) have sequenced metacercariae and sporocysts of *C. cornutus* collected from European lymnaeid and planorbid snails inhabiting sub-arctic Lake Takvatn in northern Norway but without the comparison with adults of *Cotylurus*. Recently, Gordy and Hanington (2019), in results of molecular analyses of cercariae of *Cotylurus* from freshwater ecosystems in Canada, reported the occurrence of several new lineages. Additionally, Nakao and Sasaki (2021), during molecular studies of metacercariae and sporocysts collected from *Radix auricularia* sampled from Nagayama-Shinkava River (Hokkaido, Japan), also revealed four different lineages within *Cotylurus* spp. These data strongly suggest cryptic diversity within this taxon. Regarding these facts, the issue of identity and real taxonomic diversity of *Cotylurus* tetracotyles collected from snail intermediate hosts remains almost unknown and awaits detailed studies. What is important, trematode communities in snails reflect the richness and abundance of their free-living hosts; moreover, they are suitable bioindicators of free-living diversity that allow us to assess the complex richness and full range of host-parasite interactions within ecosystems (Hechinger et al., 2007; Selbach et al., 2020; Duan et al., 2021).

In the current study, we describe the identity and molecular diversity of metacercariae of *Cotylurus* detected in several species of aquatic snails collected from two distinct localities in southern and northern Poland and analysed them on the basis of two molecular markers: 28S nuclear large ribosomal subunit gene (28S rDNA) and mitochondrial cytochrome *c* oxidase subunit 1 (CO1). Our results were supplemented by sequences obtained from adult specimens of some *Cotylurus* species, collected from avian hosts from northern Poland. On this basis, we present and discuss new data concerning the molecular diversity of *Cotylurus* spp., their phylogeny, and the implications of these findings for the structure and taxonomy of members of this genus. The presented paper is a continuation of previous work (Pyrka et al., 2021), presenting and discussing taxonomic position, molecular diversity and phylogeny of larval stages of Strigeidae occurring in leeches in Poland. It is the first comprehensive attempt to fully understand the identity and diversity of strigeid tetracotyle from snail hosts in Central Europe.

## 2 Materials and methods

### 2.1 Host sampling and parasite recovery protocols

From June to October 2020, 258 freshwater snails were collected from several sampling stations located in two distinct regions of Poland: Gdańsk Pomerania and Lower Silesia (Table 1). Sampling stations placed in Gdańsk Pomerania represent small rivers with slow-flowing waters and related drainage canals with rich vegetation, while sampling stations in Lower Silesia were located on extensive, temporarily drained fish ponds (for details see Pyrka et al., 2021). Snails were collected manually from water vegetation, littoral stones, and various objects immersed in water. Additionally, some animals were sampled from bottom detritus, using a sieve sampler. Each collected snail was transferred to a separate plastic cup with water, transported to the laboratory, stored in the fridge, and fed *ad libitum* with lettuce. Before dissection, snails were identified to the species level based on morphological characteristics provided by Jackiewicz (2000) and Piechocki and Wawrzyniak-Wydrowska (2016). Ten snail species belonging to four families (Bithynidae, Lymnaeidae, Planorbidae, Viviparidae) were identified and examined for the presence of tetracotyles (Table 1).

**Table 1:** Comparison of the prevalence and the intensity of infection of tetracotyle between two habitats.

Family/Species	Gdańsk Pomerania			Lower Silesia			Total		
	No. of necropsied/infected	Tetracotyle prevalence [%]	Tetracotyle infection intensity [min-max; mean]	No. of necropsied/infected	Tetracotyle prevalence [%]	Tetracotyle infection intensity [min-max; mean]	No. of necropsied/infected	Tetracotyle prevalence [%]	Tetracotyle infection intensity [min-max; mean]
**Bithynidae**									
*Bithynia tentaculata* (L., 1758)	23/0	–	–	–	–	–	23/0	–	–
**Lymnaeidae**									
*Ampullaceana balthica* (L., 1758)	22/6	27.3	1-74; 23	–	–	–	22/6	27.3	1-74; 23
*Lymnaea stagnalis* (L., 1758)	31/14	41.2	1-1011; 108.4	54/6	11.1	1-59; 12.5	85/20	23.5	1-1011; 79.6
*Radix auricularia* (L., 1758)	11/3	27.3	4-172; 66.3	11/9	81.8	1-159; 30.9	22/12	54.5	1-172; 41.9
*Radix labiata* (Rossmässler, 1835)	4/2	50	2-6; 4	5/0	–	–	9/2	22.2	2-6; 4
**Planorbidae**									
*Anisus spirorbis* (L., 1758)	9/0	–	–	–	–	–	9/0	–	–
*Anisus vortex* (L., 1758)	1/1	[100]	5	–	–	–	1/1	[100]	5
*Planorbarius corneus* (L., 1758)	39/0	–	–	44/10	22.7	1-41; 15.8	83/10	12.2	1-41; 15.8
*Planorbis planorbis* (L., 1758)	10/1	10	5	2/0	–	–	12/1	8.3	5
**Viviparidae**									
*Viviparus contectus* (L., 1758)	2/0	–	–	–	–	–	2/0	–	–
**TOTAL**	**142/26**	**18.3**	**1**–**1011; 71.8**	**116/25**	**21.5**	**1**–**159; 21.5**	**258/51**	**19.8**	**1**–**1011;47.1**

Before dissection, snails were anesthetised by exposing them to 2 g of crushed menthol crystals dissolved in 200 ml tap water (Krois et al., 2013). After approximately 90 min of menthol exposure, snails were removed from the menthol solution and their feet were gently scrapped by a preparation needle. Snails fully contracted into the shell or manifested life reflects after needle test were anesthetised within the next 90 minutes. Fully anesthetised snails were dissected: the shell was removed, their body cavity was opened, and all internal organs, including the inner side of shell, were examined under a stereomicroscope for the presence of parasites. Detected tetracotyles were washed in physiological salt solution and their morphology and morphometry was initially analysed. Next, collected parasites were killed in hot water, fixed and stored in 70% ethanol for further studies and molecular identification. Material for molecular analyses was randomly selected as described in previous work (Pyrka et al., 2021); in the case of low intensity of infection, all detected metacercariae were used for molecular investigations.

Additionally, several adult trematode specimens, identified on the base of their morphological features as *C. cornutus* and *C. strigeoides*, collected from gastrointestinal tract of mallard (*Anas platyrhynchos*), obtained commercially by local hunters operating on Vistula Lagoon, were used as reference material (Supplementary material, Table S1). After isolation from the intestinal tracts of the definitive avian hosts, the digeneans were rinsed in physiological salt solution and initially identified alive under the microscope then fixed in hot 70% ethanol for further morphological and molecular analyses. The selected digeneans were then stained with alcohol borax carmine, dehydrated in ethanol series, cleared in clove oil, and mounted in Canada balsam. The ecological terms used in this study are those defined by Bush et al. (1997).

### 2.2 Molecular and statistical analysis

DNA was extracted from a single, alcohol-fixed metacercariae and adult trematodes using a commercial kit (DNeasy Blood and Tissue kit; Qiagen, Hilden, Germany), according to the manufacturer's protocol. PCR amplification of nuclear 28S and the mitochondrial gene encoding CO1 was carried out using the KAPA2G Robust HotStart ReadyMix (Sigma-Aldrich, St. Louis, MO. USA), and primers were selected based on the literature and our previously study, i.e., LSU5, digl2, and 1500R for 28S rDNA and JB3, JB4.5, and CO1_Rtrema for CO1 (Pyrka et al., 2021).

The PCR results were visualised following electrophoresis in a 1% agarose gel. Obtained electrophoresis products were purified using the Exo-BAP kit (EURx) or the QIAquick Gel Extraction kit (Qiagen) when non-specific products were present. Purified products were sequenced directly in both directions using the PCR primers. Contiguous sequences were assembled using Geneious software (Geneious 9.1.8; https://www.geneious.com). The representative sequences were submitted to GenBank under accession numbers presented in Supplementary material (Table S1). The alignments including newly obtained sequences and closely related representatives of Strigeidae currently available in GenBank (Supplementary material: Table S1) were prepared using ClustalW multiple alignment implemented in MegaX (Kumar et al., 2018). Sequences of the partial 28S rDNA gene (1216 bp) and the CO1 fragment (295 bp) were aligned in two independent datasets; moreover, concatenated data (28S + CO1) was provided. Phylogenetic analyses were conducted using Bayesian inference criteria as implemented in MrBayes ver. 3.2.7 software (Ronquist et al., 2012) and were run on the three datasets (28S, CO1, and concatenated sequences of both markers) individually. The general time-reversible model with estimates of invariant sites and gamma distributed among-site variation (GTR + I + G) was identified as the best-fitting nucleotide substitution model for 28S and CO1.

The consensus trees were visualised in FigTree ver. 1.4.4 software (Rambaut, 2009) and annotated in CorelDraw® (Corel Corp., Ottawa, ON, Canada).

We also used Generalized Mixed Yule Coalescent (GMYC) model analysis as a tool for species delimitation (Pons et al., 2006; Fujisawa and Barraclough, 2013). This method works for a single locus tree; we used the CO1 sequence to construct an ultrametric tree with BEAST v. 2.4.4 (Bouckaert et al., 2014). Prior to analysis, the alignment was collapsed to unique haplotypes and the outgroup was removed. Thus, the GMYC analysis contained 37 haplotypes, the nucleotide substitution model was set to HKY + G, and we used the coalescent model with constant population size (which is the most appropriate for modelling the relationships among individuals from the same species) with a strict clock. GMYC analysis was done in R software (R v. 4.0.2; R Foundation for Statistical Computing, Vienna, Austria) with the following packages: “ape”, “paran”, “rncl”, and “splits”.

To assess the significance of locality- and host-dependent variability in tetracotyle infection parameters, we use standard statistical tools, such as the chi-square test (for prevalence) and the Mann-Whitney test (for mean intensity), implemented in Statistica 13 software.

## 3 Results

### 3.1 Parameters of infection

Of the 258 analysed snails, metacercariae of the tetracotyle type were detected in the body cavity and tissues of the hepatopancreas in 51 specimens (overall prevalence 19.8%). We did not observe significant differences in prevalence between localities, i.e., in Gdańsk Pomerania, the prevalence was 18.3%, while in Lower Silesia, it was 21.5% (χ^2^ = 0.49, p = 0.481; Table 1). On the other hand, regarding pooled data, we have noticed significant host-dependent differences in infection rate (χ^2^ = 30.91, p < 0.001); the highest prevalence of metacercariae was observed in *Radix auricularia* (12 infected among 22 investigated, 54.5%), *Ampullaceana balthica* (6 infected among 22 examined, 27.3%), *Lymnaea stagnalis* (20 infected among 85 investigated, 23.5%), and *Radix labiata* (2 infected among 9 examined, 22.2%). Only one specimen of *Anisus vortex* was examined, and it was infected (Table 1). The lowest prevalence recorded in the present study was in *Planorbis planorbis* (1 infected among 12 examined, 8.3%), then *Planorbarius corneus* (10 infected among 83 investigated, 12.2%). Three snail species (*Bithynia tentaculata*, *Anisus spirorbis*, *Viviparus contectus*) were not infected by tetracotyles (Table 1). In some snail species, we observed clearly expressed locality-dependent variability in prevalence; in *L. stagnalis*, the prevalence of tetracotyles was much higher in specimens collected from Gdańsk Pomerania than in Lower Silesia (41.2% vs. 11.1%, χ^2^ = 12.01, p < 0.001). The opposite trend was observed in *R. auricularia* (27.3% vs. 81.8%, χ^2^ = 6.60, p < 0.010) (Table 1). In the case of *Planorbarius corneus*, only specimens from Lower Silesia were infected, while in *A. balthica* and *Planorbis planorbis*, the occurrence of tetracotyles was recorded exclusively in snails from Gdańsk Pomerania (Table 1).

The relatively high mean intensity (71.8) was recorded among snails from Gdańsk Pomerania in comparison to snails from Lower Silesia (21.5; Z = 2.14, p = 0.032) (Table 1). Analysing combined data for both localities and particular host species, the highest mean intensity of tetracotyles was recorded in *L. stagnalis* (79.6), *R. auricularia* (47.9), *A. balthica* (23), and *Planorbarius corneus* (15.8). The lowest mean intensity was noted in *R. labiata* (4), before *A. vortex* and *Planorbis planorbis* (5); however, only single specimens of these hosts were infected (Table 1). Similarly, as in the case of prevalence, in some snail species, we observed strong locality-dependent variability in mean intensity; in *L. stagnalis,* the mean intensity was significantly higher in the sample collected from Gdańsk Pomerania (108.4) than in Lower Silesia (12.5; Z = 2.883; p < 0.001), similarly as in the case of *R. auricularia* (66.3 vs. 30.9; Z = -1.838; p = 0.05) (Table 1).

### 3.2 Molecular identification and phylogenetic analyses

The conducted analysis based on BLAST (Basic Local Alignment Search Tool) comparison of 28S rDNA sequences obtained from collected metacerariae derived from snails indisputably confirmed their taxonomic affiliation as *Cotylurus* spp.

Since previous phylogenetic analyses (Pyrka et al., 2021) indisputably revealed the validity of genus *Cotylurus* and its separate position within Strigeidae, the presented work focuses exclusively on identifying taxonomic position of detected tetracotyles from snails and their associations with adult trematodes from avian definitive hosts*,* as well as molecular diversity and phylogenetic relationships on the basis of recently obtained isolates and limited data available in GenBank. Regarding fact, that comprehensive molecular studies of *Cotylurus* spp. are rather rare in the literature, the structure of our datasets was determined mainly by the availability of sequences from previously published European isolates. For this reason, the sequences of *Cotylurus* published previously by Heneberg et al. (2018) and Pyrka et al. (2021) constitute a significant part of the comparative material necessary for establishing the taxonomic position of the recently collected and analysed materials, thus CO1 alignments used for our phylogenetic analyses were trimmed to the length used in the cited papers (295 bp). In the present study, we performed three independent phylogenetic analyses based on the 28S rDNA (39 sequences, including 30 new), CO1 (55 sequences, including 40 new), and combined analysis of both markers (based on 28 sequences). In our opinion, the topology of the trees based on 28S rDNA and CO1 does not differ significantly from the concatenated tree, however, regarding informativeness of obtained results as a basis for phylogenetic and taxonomic considerations, we selected the results received from the concatenated analysis. We present a detailed description of observed relationships between particular taxa based on the combined tree, but the emerging discrepancies between the markers were also presented and discussed. However, to more fully illustrate and distinguish potential species, GMYC analysis (based on CO1 marker) was also performed. This analysis revealed the presence of 11 operational taxonomical units (OTU) among 37 unique haplotypes. The results obtained in GMYC analysis have been implemented to each phylogenetic tree as “lineages”, annotated by Roman numerals.

**Fig. 1:**
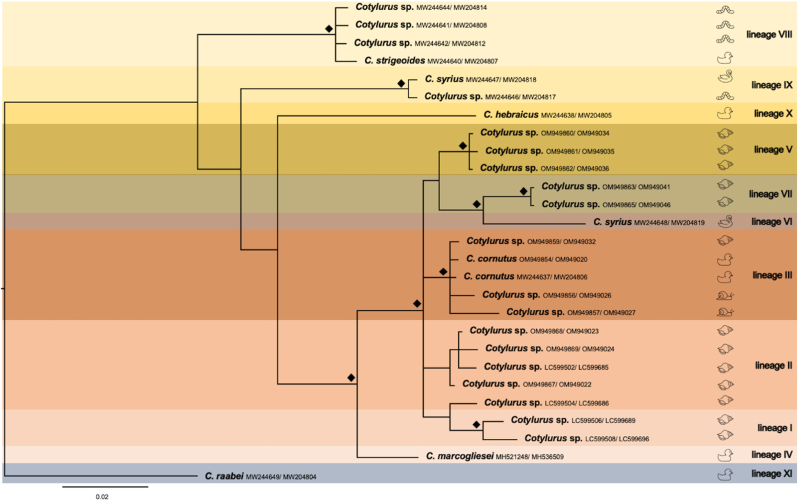
The phylogenetic relationships within genus *Cotylurus* based on the concatenated COI mtDNA and 28S rDNA markers. The analysis was performed by the use of Bayesian inference, diamond symbol indicates posterior probability greater than 90%.

**Fig. 2:**
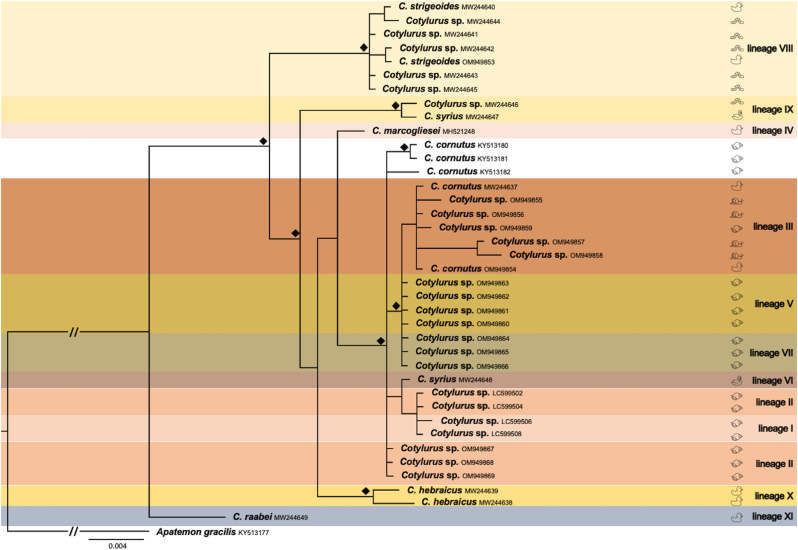
The phylogenetic relationships within the genus *Cotylurus* based on 28S rDNA marker. The analysis was performed by the use of Bayesian inference, diamond symbol indicates posterior probability greater than 90%.

**Fig. 3:**
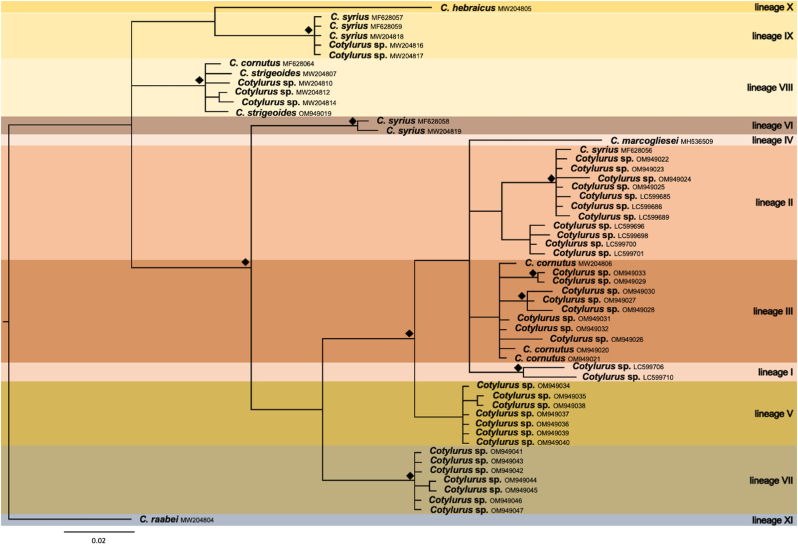
The phylogenetic relationships within the genus *Cotylurus* based on COI mtDNA marker. The analysis was performed by the use of Bayesian inference, diamond symbol indicates posterior probability greater than 90%.

**Fig. 4:**
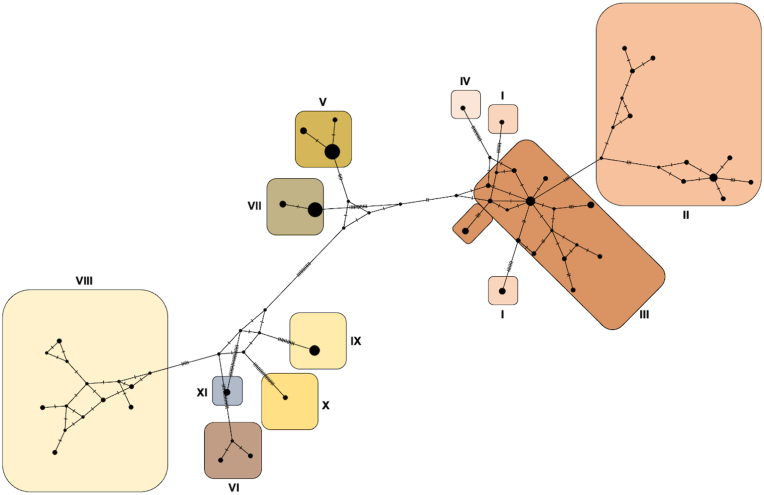
A median-joining network of COI haplotype of *Cotylurus*. Each circle represents a unique haplotype where the diameter is proportional to the number of DNA sequences represented.

Observed differences between all three obtained phylogenetic trees (Fig. 2, Fig. 3, Fig. 4) mainly concerned the number of sequences used for the analysis (according to their availability), the internal structure of the “*C. cornutus* sensu lato” clade, and the variable position of *C. hebraicus* Dubois, 1934 and *C. syrius* B. Indisputably, the presented concatenated phylogenetic tree revealed the separate and distinct position of *Cotylurus raabei* (Bezubik, 1958) [lineage XI] (Fig. 1) far from other clades of *Cotylurus*. Interestingly, strongly supported (100%) and clearly distinct positions within other *Cotylurus* taxa revealed two clades utilising leeches as intermediate hosts. The first contains isolates of *C. strigeoides* (“*C. strigeoides”* [lineage VIII]) obtained from an adult trematode sampled from an avian definitive host *(A. platyrhynchos*) and tetracotyle larvae collected from leeches, while the second clade [lineage IX], described as *C. syrius* A, included isolates of *C. syrius* obtained both from avian definitive (*Cygnus olor*) and leech intermediate (*Haemopis sanguisuga*) hosts (Fig. 1). Other separate branches led to two single species: *C. hebraicus* [lineage X] and *C. marcogliesei* [lineage IV], which surround a large group of diverse sequences representing the “*C. cornutus* sensu lato” clade (Fig. 1). The position of *C. hebraicus* differs significantly in both single-marker trees. In the phylogram based on CO1 sequences, *C. hebraicus* formed a sister clade to *C. syrius* A (Fig. 2), while in 28S rDNA analysis, this lineage is basal to the “*C. cornutus* sensu lato” branch (Fig. 3). Unfortunately, the resolution of the latter group on the concatenated tree is not very high due to a limited number of available sequences; however, the most complete picture of diversity within the “*C cornutus* sensu lato” clade was demonstrated by the CO1 phylogram, which revealed the existence of several lineages within (Fig. 2). One of these clades [lineage II] consists of two branches. One branch consists of clustered isolates determined by Heneberg et al. (2018) as *C. syrius* (*C. syrius* C) obtained from *Cy. olor* from the Czech Republic; sequences derived from metacercariae described by Nakao and Sasaki (2021) as *Cotylurus* sp. A collected from *R. auricularia* from Japan, and four isolates generated in this study from tetracotyles collected from lymnaeid and planorbid snails (Fig. 2). The second branch within lineage II consists of sequences obtained from sporocysts sampled from snails (*R. auricularia*) in Japan, identified by Nakao and Sasaki (2021) as *Cotylurus* sp. B (Fig. 2). The large neighbouring clade [lineage III] within the “*C. cornutus* sensu lato” branch is the most diverse and clustered. It consists of several recently obtained sequences of metacercariae derived from lymnaeid and planorbid snails, two new isolates obtained from adult trematodes determined as *C. cornutus* collected from *A. platyrhynchos* from Vistula Lagoon, and a previously published isolate of *C. cornutus* collected from Gdańsk Pomerania (Fig. 2). This lineage, in our opinion, reflects molecular variation within the “*C. cornutus* sensu stricto” taxon (Fig. 2). Another group [lineage I] consist of two sequences, described as *Cotylurus* sp. C and D, obtained from sporocysts collected from *R. auricularia* in Japan (Fig. 2). The next two clearly separate clades [lineages V and VII] are formed by newly obtained sequences of metacercariae; however, these lineages clearly revealed well expressed host- and locality-dependent patterns. The first of these groups (lineage V in Fig. 2) revealed significant locality-dependent relationships. In this lineage, the sequences recently obtained from tetracotyles from Lymnaeidae and Planorbidae intermediate hosts but collected only in Gdańsk Pomerania (Fig. 3) were clustered. In the last group (lineage VII in Fig. 2), we observed strongly expressed host-dependent relationships; this lineage consists of isolates from tetracotyles collected only from Lymneidae snails, both from Gdańsk Pomerania and Lower Silesia.

Phylogenetic analysis based on a fragment of the CO1 gene fully confirmed (revealed based on partial 28S rRNA analysis) an inconsistent position of *C. syrius*. However, phylogeny based on mitochondrial locus revealed the existence of three (not two, as resulting from phylogeny based on 28S rDNA) well-supported and separate lineages of *C. syrius* (see Fig. 2, Fig. 3 for comparison). The first of these lineages (“*C. syrius* A” lineage - clade IX) is formed by several isolates of *C. syrius* obtained from definitive avian hosts from the Czech Republic and Poland and tetracotyle metacercariae collected from leeches (*H. sanguisuga*) from Poland (Fig. 2). The second lineage of *C. syrius* (“*C. syrius* B” lineage - clade VI) consists of two sequences obtained from adult trematodes determined as *C. syrius* and sampled from avian host *Cy. olor* from the Czech Republic and Poland (Fig. 2). The third lineage of *C. syrius* (“*C. syrius* C” lineage - clade II) is represented by sequences obtained from a definitive avian host (*Cy. olor*) from the Czech Republic. Its phylogeny is based on the analysis of sequences of the CO1 gene located in the “*C. cornutus* sensu lato” branch and clustered with several sequences derived from metacercariae from Japan described by Nakao and Sasaki (2021) as *Cotylurus* sp. A and four isolates recently obtained from tetracotyle metacercariae collected from lymneid and planorbid snails (Fig. 2).

### 3.3 Haplotype analysis

In general, the median-joining haplotype network demonstrated similar findings to those derived from phylogeny based on CO1 sequences, concatenated (28S + CO1) data, and GMYC analysis (Fig. 4). Most taxa revealed in these phylogenies can be easily distinguished in the obtained haplotype network; however, in part of the network, the imperfectly defined group that represents sequences derived from the “*C. cornutus* sensu stricto” branch can be observed (Fig. 4). Most of these sequences clustered in this area of the network differ in 1–2 nucleotides. Both the structure of this part of the network and the absence of a dominant haplotype may indicate either a species characterised by a high level of variability (not found in other distinguished *Cotylurus* lineages) or the presence of a complex of several poorly differentiated species-level lineages.

### 3.4 Nucleotide pairwise comparison within *Cotylurus* spp

The overall interspecific divergence of analysed representatives of *Cotylurus* based on 28S rDNA alignment was 0.0%–3.13% (0–38 bp out of 1216 bp). However, when *C. raabei* was removed from this comparison, these values were significantly lower (0.0%–2.47%, 0–30 bp). Divergence within the “*C. strigeoides*” lineage was between 0.0% and 0.25%, while sequences of adult digeneans and larvae clustered within the “*C. cornutus* sensu lato” branch showed divergence in the range of 0.0%–1.23%.

Additionally, pairwise nucleotide comparisons of CO1 sequences within analysed *Cotylurus* spp. lineages are also provided. There were no intraspecific differences between the isolates of *C. syrius* A [lineage IX], while differentiation ranging between 1.02% and 1.70% was noted among sequences clustered within *C. strigeoides* (lineage VIII), and the overall interspecific divergence within sequences of the “*C. cornutus* sensu lato” group was 0%–7.8%. However, within the lineages of the “*C. cornutus* s.l.” clade, the maximum value of intraspecific nucleotide differences was as follows: for lineage II - 4.07%, for lineage III - 2.37%, and for lineages V and VII - 0.34%. For comparison, *C. raabei* and *C. strigeoides* differ by 8.14%, while *C. raabei* and *C. cornutus* differ by 9.83%.

## 4 Discussion

The results obtained in the present study give a new perspective on the perception of the diversity of larvae of strigeid trematodes occurring in freshwater snails. The current work indisputably revealed that all tetracotyles collected from snail intermediate hosts represent members of the genus *Cotylurus*. Provided molecular analysis discovered their unexpectedly high molecular diversity with clearly expressed evidence of cryptic diversity and the existence of novel-species lineages. Our findings shed new light on the phylogenetical and ecological relationships within *Cotylurus*.

In the current work, the observed overall prevalence of tetracotyles of *Cotylurus* spp. detected in snails (19.8%) was relatively low compared to that reported in other studies. However, in most available reports, only *Lymnaea* snail hosts were examined for the presence of tetracotyles. For example, Zajíček (1971) detected tetracotyles in 89.8% of investigated lymnaeid snails (*Lymnaea auricularia*, *L. stagnalis*) from a territory of former Czechoslovakia. Similar results were obtained by Yoder and Coggins (1998), indicating the prevalence of tetracotyles of *C. flabelliformis* Van Haitsma, 1931in *L. stagnalis* from Southeastern Wisconsin varied between 92.8% and 78.6%, depending on the year of the study. Ulmer (1957) detected tetracotyles of *C. flabelliformis* in over 99% of analysed specimens of *L. reflexa*. Arustanov (1970) stated that, in some sampling stations located in the waters of Amu-daria River and southern Aral Sea, the prevalence of tetracotyles within a population of water snails often exceeded 50%. However, the prevalence observed in the current research should be recognised as relatively low, as only one species (*R. auricularia*) exceeded 50% (Table 1). Interestingly, during investigations of patterns of trematode parasitism in lymnaeid snails (*L. stagnalis*, *L. peregra*) from three freshwater lakes in northern and central Finland, Väyrynen et al. (2000) revealed that the prevalence of tetracotyles varied significantly between sampling stations. In some lakes, tetracotyles were absent, while in the others, prevalence reached 37.8% and 64.0%, respectively. Our results confirm this phenomenon of significant locality-dependent variability of tetracotyle infection, i.e., the prevalence of tetracotyles in *L. stagnalis* depends significantly on the study site and ranged from 11.1% (Lower Silesia) to 41.2% (Gdańsk Pomerania). This is similar to that of *R. auricularia*, ranging from 27.3% (Gdańsk Pomerania) to 81.8% (Lower Silesia), but specimens of *R. labiata* were found infected (50%) only in Gdańsk Pomerania (Table 1). In the case of all infected species, mean intensity was always significantly higher in snails from Gdańsk Pomerania (Table 1). Our findings supplement the data concerning the specificity of tetracotyles in relation to their snail host. Up to date, according to literature data, tetracotyles were detected mainly in Lymnaeidae (Wikgren, 1956; Arustanov, 1970; Nakao and Sasaki, 2021) and Planorbidae (Basch, 1969; Soldánová et al., 2017; López-Hernández et al., 2019), and only rarely in Bithyniidae (Wikgren, 1956; Serbina, 2014) or Semisulcospiridae (Urabe, 2001 and 2003). From an ecological point of view, it is interesting that the prevalence of *Cotylurus* tetracotyles within the population of leeches collected in the same sampling sites (7.6% in Gdańsk Pomerania and 6.0% in Lower Silesia) presented in previous work (Pyrka et al., 2021), were much lower than the observed prevalence of tetracotyles in snails (18.3% in Gdańsk Pomerania and 21.5% in Lower Silesia). We cannot exclude observed differences resulting from different principles of transmission success between particular species of *Cotylurus* using snails and leeches as second intermediate hosts. However, the influence of other factors is also possible. This situation raises several key questions concerning the origins and effectiveness of different life strategies within particular *Cotylulus* species, which can be resolved by further studies based on the molecular elucidation of *Cotylurus* life cycles and exploration of a wide range of host-parasite interactions within particular ecosystems.

One of the most important achievements of the present work is solving the mysterious problem of the taxonomic position of tetracotyles occurring in snails and leeches. We confirmed suggestion from a previous paper (Pyrka et al., 2021), concerning the existence of two separate ecological lineages within species of *Cotylurus*. The first of these lineages, with contemporary evidence of two species (*C. strigeoides* and *C. syrius* A), were based on molecular analysis of tetracotyles who used leeches as a host for metacercariae. In the second lineage (currently composed of species-level lineages grouping within “*C. cornutus* sensu lato” branch and *C. syrius* B), tetracotyle occur exclusively in snail intermediate hosts. In obtained phylogenies based on all analysed loci, isolates obtained from adult trematodes and/or tetracotyles of *Cotylurus* spp. with confirmed invasive stages in leeches (*C. hebraicus*, *C. strigeoides* and *C. syrius* A) clustered in different, clearly separate branches of the obtained tree than the species-level lineages of *Cotylurus* with tetracotyle in snails (“*C. cornutus* sensu lato” branch and *C. syrius* B), which clearly indicate direct relationships between the existence of these ecological lineages and phylogeny of *Cotylurus*. As mentioned above, over the years, tetracotyles widely recorded in snails and leeches in Europe had been classified as *Tetracotyle typica* de Fillippi, 1855, but their true taxonomic position has been not established in detail. Generally, the adult stage of *T. typica* collected from both groups of intermediate hosts has been identified as *C. cornutus* (e.g., Szidat, 1929; Timon-David, 1943; Dobrowolski, 1958; Sudarikov, 1959 and references therein; Spelling and Young, 1986). Regarding literature data, leeches have been recognised as hosts for metacercariae of four species of *Cotylurus* detected in Europe: *C. cornutus*, *C. hebraicus*, *C. strigeoides* and *C. szidati* Zazornova, 1991 (Zajíček, 1971; Zazornova, 1987, 1991, 1993; Sudarikov et al., 2002). Tetracotyles of three of these species (*C. cornutus*, *C. hebraicus*, *C. strigeoides*) were also recorded in snails (Zajíček, 1971; Fried et al., 1978; Zazornova, 1987, 1993; Sudarikov et al., 2002). In this light, historical reports concerning the occurrence of tetracotyles of *C. cornutus* in leeches and *C. strigeoides* in snails, not confirmed by molecular analysis or feeding experiments, should be treated with caution. Similarly surprising are data presented by Fried et al. (1978), who collected tetracotyles from snail *Physa heterostropha* and raised them in chicks to obtain trematode specimens identified as *C. strigeoides*. These data may indicate that *C. strigeoides* may consist of a *de facto* complex of cryptic species, characterised by similar morphology but with different life cycles. In diversified material collected from leeches and snails sampled from the same localities in Poland (Pyrka et al., 2021; this study), the occurrence of tetracotyles of *C. strigeoides* was detected and confirmed by molecular methods only in leeches.

As suggested previously by Pyrka et al. (2021), another interesting issue concerning the diversified structure and molecular variability of *C. strigeoides* is the surprising position of isolate derived by Heneberg et al. (2018) from common teal *Anas crecca* from the Czech Republic and described as *C. cornutus* (MF628064) (Fig. 3). Excluding the possibility of incorrect determination of trematode specimens, the position of this isolate can result from high morphological variability of adult specimens of *C. strigeoides*, signaled previously by Zajíček (1971) and Zazornova (2004). In the case of poorly or undeveloped vitellaria in the forebody (prosoma according to Achatz et al., 2022), *C. strigeoides* can be easily confused with *C. cornutus* and *C. brevis* Dubois et Rausch, 1950, which has been emphasised earlier by Zajíček (1971). Considering unresolved morphological variability of *C. strigeoides* and the dependencies underlying it, very interesting are data provided by Zazornova (1987), who investigated the life cycle of *C. hebraicus*. She detected metacercariae of this species in leech (*Erpobdella octoculata*, *Glossiphonia concolor*, *Haemopis sangiusuga*) and snail (*Physa fontinalis*) intermediate hosts, both in natural and experimental infections. Unfortunately, the validity of these determinations, based exclusively on morphological and morphometric features of tetracotyles, was not confirmed by any experimental infection of a definitive avian host. Regarding an extremely high level of homogeneity in the morphology of tetracotyles and likely narrow host specificity to second intermediate hosts, it cannot be excluded, that Zazornova (1987) analysed *de facto* morphologically similar tetracotyles of two different species, utilising both leeches and snails as hosts. In this light, the issue of morphological variability and molecular diversity within both tetracotyles and adult specimens of *C. strigeoides* and *C. hebraicus* remains unexplained and awaits further studies.

Another interesting issue concerning the obtained results is attempting to resolve the unclear and composite structure of *C. syrius* reported previously by Heneberg et al. (2018) and Pyrka et al. (2021). Indisputably, the currently obtained phylogenies clearly confirmed the existence of three separate molecular lineages within *C. syrius* (identified on the base of morphology)*,* trematodes collected from mute swans (*Cygnus olor*). It seems that “*C. syrius*” represents at least three morphologically indistinguishable but molecularly and likely ecologically distinct species: *C. syrius* A, with confirmed occurrence of tetracotyles in leeches; *C. syrius* B, most likely with metacercariae in snails; and *C. syrius* C, molecularly representing *C. cornutus*, also with tetracotyles in snails. Final confirmation of suggested life cycles of *C. syrius* B and C requires further detailed studies and molecular confirmation. What is important, except for the occurrence of cryptic species within *C. syrius* and *C. cornutus*, and confirmation of the existence of crucial differentiation of life cycles within morphologically indistinguishable taxa, our findings show a clearly direct link between variability of life cycles and phylogeny within *Cotylurus*. The consequences of this fact cannot be overestimated; however, it sheds new light on the perception of the phenomenon of species distinctiveness and evolution of life cycles within Strigeidae.

Obtained results also provide several new and important insights into the molecular diversity of *Cotylurus* metacercariae occurring in freshwater snails in central Europe, as well as *Cotylurus* diversity as a whole. In literature data, the unclear taxonomic status of the type-species of *Cotylurus*, *C. cornutus*, has been widely emphasised. This species has been described by Rudolphi (1809) based on specimens collected from European golden plover (*Pluvialis apricaria*) from Central Europe under the name *Amphistoma cornutum*. Further, Szidat (1928) placed it as a type-species within the newly erected genus *Cotylurus*. Over the years, some authors recognised *C. cornutus* as a complex of species (Zazornova, 2004, 2010) with a wide range of avian definitive hosts and a large number of synonyms resulting from great morphological variability among adult trematodes (for details, see Zazornova, 2004, 2010). Explaining sources of these variabilities within trematodes identified as *C. cornutus*, Dubois and Rausch (1950) described a new species, *Cotylurus brevis*. Results of examination of various individuals from avian definitive hosts convinced Dubois and Rausch (1950) that *C. brevis* had often been confused with *C. cornutus*, e.g., authors stated that resolved experimentally by Timon-David (1943) fragment of life cycle (from tetracotyle to adult worm) of *C. cornutus*, probably concerning *C. brevis*. Unfortunately, the topic of the potential existence of cryptic species within *C. cornutus* was not addressed in this work. Earlier,Matthias (1925) investigated the life cycle of *Strigea tarda*. This species was considered a synonym of *C. cornutus* by Szidat (1928). Later, Dubois (1953) recognised this species as a synonym of *C. brevis*. Nasir (1960) considered that *C. brevis* morphologically is very closely related to *C. cornutus,* thus these taxa can be very easily confused. Dubois (1968), in his monograph, finally recognised that *C. cornutus* is a characteristic parasite of Charadriformes, while *C. brevis* occurs mainly in Anatidae. According to Sudarikov (1984), these assumptions required verification, similar to morphological variability among specimens of *C. cornutus* from a wide range of avian final host species.

Previous works, based on molecular analysis of adult *Cotylurus* trematodes (Heneberg et al., 2018; Pyrka et al., 2021), indisputably revealed the unclear phylogenetic position of specimens collected from anatid birds and identified on base of morphological features as *C. cornutus.* And tthese results confirmed earlier suggestions of Zazornova (2004, 2010) regarding *C. cornutus* as a complex of species. Our results also revealed unexpectedly high diversity within sequences received both from adult flukes determined as *C. cornutus* and tetracotyles from snails recognised as *Cotylurus* spp. from the large “*C. cornutus* sensu lato” branch. Based on evaluation of mitochondrial locus (CO1), concatenated data (28S rDNA + CO1), and conducted GMYC analysis, the recently acquired isolates of tetracotyles collected from snails in Poland clearly constitute four distinct species-level lineages: two of these lineages [II and III] are strongly diversified and reflect molecular variability within lineages related to *C. cornutus* with indisputably expressed evidence of cryptic diversity. Two other less diversified lineages [V and VII] likely represent additional species-level lineages within *Cotylurus* with unknown identity. In our opinion, the occurrence of these two lineages within the “*C. cornutus* sensu lato” branch can reflect heterogeneity resulting from the unclear taxonomic position of *C. cornutus* and its far from final established structure. In our opinion all isolates obtained from adult trematodes identified as *C. cornutus* clustering within lineage III consist of representatives of “*C. cornutus* sensu stricto”. The “*C. cornutus* sensu stricto” lineage is characterised by a high level of variability not observed in other *Cotylurus* species-level lineages obtained in the presented phylogenies. Haplotype analyses clearly revealed that “*C. cornutus* sensu stricto” lineage is characterised by the occurrence of several equivalent haplotypes without indisputable dominance of any of them. In our opinion, this situation can result from the presence of a complex of several inadequately differentiated species or indicate a species characterised by a high level of variability. We cannot exclude the idea that the first mentioned lineage within the “*C. cornutus* sensu lato” branch may consist of *C. cornutus* related with charadriform birds as final hosts and/or representatives of *C. brevis*. However, occurrence within this lineage isolates described as *C. syrius* (*C. syrius* C, MF628056), which species is morphologically more similar to *C. cornutus* than to *C. brevis*, may suggest the first of these possibilities. Thus, the real taxonomic status of typical species of genus *Cotylurus*, *C. cornutus,* from a wide range of final hosts and its phylogenetic relationships with *C. brevis* are still far from final establishing and require further detailed analysis of specimens collected both from anseriform and charadriform final hosts. Moreover, detailed analyses of their life cycles and morphology of the larval stages (cercariae) may also be necessary to solve this issue.

Recently obtained data have also revealed new and interesting data concerning species richness within the genus *Cotylurus*. GMYC analysis based on the CO1 locus revealed the presence of 11 OTU among 37 unique haplotypes, clearly confirming the validity of some species (*C. hebraicus*, *C. raabei*, *C. marcoglisei*, *C. strigeoides*) while showing the presence of cryptic diversity within the others (3 species-level lineages within specimens morphologically identified as *C. syrius*, nothing less than 4 species-level lineages within the branch defined as “*C. cornutus* s.l*.*“). Regarding molecularly confirmed data from Poland, *Cotylurus* fauna consists of nine species-level lineages (“*C. cornutus* sensu lato” branch with four lineages: *C. hebraicus*, *C. raabei*, *C. strigeoides*, and *C. syrius* A and B), while based on morphological criteria, five species of *Cotylurus* were identified in avian hosts (*C. cornutus*, *C. hebraicus*, *C. raabei*, *C. strigeoides*, and *C. syrius*). These facts clearly proved that the real diversity of *Cotylurus* is still far from understood.

In conclusion, our data shed light on the issue of the unexpectedly complex and multi-faceted structure of the genus *Cotylurus* with clear evidence of cryptic diversity and the occurrence of several species-level lineages. We confirmed the existence of two divergent phylogenetical and ecological lineages within *Cotylurus*, differing significantly in life history strategies. Moreover, the presented paper created a framework for further studies of *Cotylurus* diversity, as well as the diversity of Strigeidae as a whole, and clearly revealed the necessity of further studies concerning diversity and phylogenetic relationships within genus *Cotylurus*. In our opinion, denser taxon sampling and greater geographic and host coverage (based on comparative analyses of material collected from intermediate and final hosts) may bring several new discoveries, changing our perception of the diversity and ecology of *Cotylurus*, as well as other Strigeidae.

## Declarations of competing interest

The authors declare that they have no competing interest
