# Supplementary material for: Life history strategies of Cotylurus spp. Szidat, 1928 (Trematoda, Strigeidae) in the molecular era – Evolutionary consequences and implications for taxonomy
Source: Int J Parasitol Parasites Wildl. 2022 Jun 11;18:201–11. doi: 10.1016/j.ijppaw.2022.06.002 (PMC9207058; doi:10.1016/j.ijppaw.2022.06.002)
Supplement: Multimedia component 1 [file mmc1.docx]

| Digenean taxa | Host species and geographic origin | Hosts | GenBank Nos | | References |
| --- | --- | --- | --- | --- | --- |
|  |  |  | CO1 | 28S |  |
| *Cotylurus strigeoides* Dubois, 1958 | *Anas platyrhynchos*  Poland | B | S9 /MW204807 | S9 /MW244640 | Pyrka et al. 2021 |
| *Cotylurus* sp. | *Haemopis sanguisuga*  Poland | L | PG3/ MW204814 | PG3/ MW244644 | Pyrka et al. 2021 |
| *Cotylurus* sp. | *Haemopis sanguisuga*  Poland | L |  | PS1/ MW244641 | Pyrka et al. 2021 |
| *Cotylurus* sp. | *Erpobdella octoculata*  Poland | L | PS10/ MW204812 | PS10/ MW244642 | Pyrka et al. 2021 |
| *Cotylurus* sp. | *Haemopis sanguisuga*  Poland | L | PS5/ MW204810 | PS5/ MW244643 | Pyrka et al. 2021 |
| *Cotylurus cornutus* (Rudolphi, 1808) | *Anas platyrhynchos*  Poland | B | S10/ MW204806 | S10/ MW244637 | Pyrka et al. 2021 |
| *Cotylurus syrius* Dubois, 1934 | *Cygnus olor*,  Poland | B | LD/ MW204819 | LD/ MW244648 | Pyrka et al. 2021 |
| *Cotylurus hebraicus* Dubois, 1934 | *Fulica atra*  Poland | B | 1443/ MW204805 | 1443/ MW244638 | Pyrka et al. 2021 |
| *Cotylurus hebraicus* Dubois, 1934 | *Fulica atra*  Poland | B | - | 1492/ MW244639 | Pyrka et al. 2021 |
| *Cotylurus raabei* (Bezubik, 1956) | *Anas platyrhynchos*  Poland | B | S2/ MW204804 | S2/ MW244649 | Pyrka et al. 2021 |
| *Apatemon gracilis* (Rudolphi, 1819) | *Gasterosteus aculeatus*  Norway | S | - | KY513177 | Soldánová et al. 2017 |
| *Cotylurus syrius* Dubois, 1934 | *Cygnus olor*  Czech Republic | B | MF628056 | - | Heneberg et al. 2018 |
| *Cotylurus syrius* Dubois, 1934 | *Cygnus olor*  Czech Republic | B | MF628057 | - | Heneberg et al. 2018 |
| *Cotylurus syrius* Dubois, 1934 | *Cygnus olor*  Czech Republic | B | MF628059 | - | Heneberg et al. 2018 |
| *Cotylurus cornutus* (Rudolphi, 1808) | *Anas crecca*  Czech Republic | B | MF628064 | - | Heneberg et al. 2018 |
| *Cotylurus syrius* Dubois, 1934 | *Cygnus olor*  Czech Republic | B | MF628058 | - | Heneberg et al. 2018 |
| ***Cotylurus strigeoides* Dubois, 1958** | ***Anas platyrhynchos***  **Poland** | **B** | **GDK_2_2/ OM949019** | **GDK_2_2/ OM949853** | **This study** |
| *Cotylurus* sp. | *Haemopis sanguisuga*  Poland | L |  | PG8_C_Hs/ MW244645 | Pyrka et al. 2021 |
| Cotylurus sp. | Haemopis sanguisuga  Poland | L | PG9/ MW204817 | PG9_C_Hs/ MW244646 | Pyrka et al. 2021 |
| *Cotylurus syrius* Dubois, 1934 | *Cygnus olor*  Poland | B | LK/ MW204818 | LK/ MW244647 | Pyrka et al. 2021 |
| *Cotylurus marcogliesei* Locke, Van Dam, Caffara, Alves Pinto, Lopez-Hernandez & Blanar, 2018 | *Lophodytes cucullatus*  Canada | B |  | MH521248 | Locke at al. 2018 |
| ***Cotylurus* sp.** | ***Planorbarius corneus***  **Poland** | **S** |  | **ZR_S_160/ OM949855** | **This study** |
| ***Cotylurus* sp.** | ***Planorbarius corneus***  **Poland** | **S** | **ZR_S_224/ OM949026** | **ZR_S_224/ OM949856** | **This study** |
| ***Cotylurus* sp.** | ***Lymnaea stagnalis***  **Poland** | **S** | **BS_S_7wr/ OM949032** | **BS_S_7wr/ OM949859** | **This study** |
| ***Cotylurus* sp.** | ***Lymnaea stagnalis***  **Poland** | **S** | **BS_S_11wr/ OM949041** | **BS_S_11wr/ OM949866** | **This study** |
| ***Cotylurus* sp.** | ***Planorbarius corneus***  **Poland** | **S** | **ZR_S_226/ OM949027** | **ZR_S_226/ OM949857** | **This study** |
| ***Cotylurus* sp.** | ***Planorbarius corneus***  **Poland** | **S** |  | **ZR_S_227/ OM949858** | **This study** |
| ***Cotylurus cornutus* (Rudolphi, 1808)** | ***Anas platyrhynchos***  **Poland** | **B** | **GDK_2_5/ OM949020** | **GDK_2_5/ OM949854** | **This study** |
| ***Cotylurus* sp.** | ***Lymnaea stagnalis***  **Poland** | **S** |  | **BS_G_63/ OM949863** | **This study** |
| ***Cotylurus* sp.** | ***Lymnaea stagnalis***  **Poland** | **S** | **BS_G_7b/ OM949036** | **BS_G_7b/ OM949862** | **This study** |
| ***Cotylurus* sp.** | ***Lymnaea stagnalis***  **Poland** | **S** | **BS_G_6/ OM949035** | **BS_G_6/ OM949861** | **This study** |
| ***Cotylurus* sp.** | ***Lymnaea stagnalis***  **Poland** | **S** | **BS_G_3a/ OM949034** | **BS_G_3a/ OM949860** | **This study** |
| ***Cotylurus* sp.** | ***Radix balthica***  **Poland** |  |  | **BJ_G_113/ OM949864** | **This study** |
| ***Cotylurus* sp.** | ***Radix auricularia***  **Poland** | **S** | **BU_S_169a/ OM949046** | **BU_S_169a/ OM949865** | **This study** |
| *Cotylurus* sp. | *Radix auricularia*  Japan | S |  | LC599502 | Nakao and Sasaki, 2021 |
| *Cotylurus* sp. | *Radix auricularia*  Japan | S |  | LC599504 | Nakao and Sasaki, 2021 |
| *Cotylurus* sp. | *Radix auricularia*  Japan | S |  | LC599506 | Nakao and Sasaki, 2021 |
| *Cotylurus* sp. | *Radix auricularia*  Japan | S |  | LC599508 | Nakao and Sasaki, 2021 |
| ***Cotylurus* sp.** | **Radix labiate**  **Poland** | **S** | **RL_G_102a/ OM949022** | **RL_G_102a/ OM949867** | **This study** |
| ***Cotylurus* sp.** | ***Radix auricularia***  **Poland** | **S** | **BU_S_149/ OM949023** | **BU_S_149/ OM949868** | **This study** |
| ***Cotylurus* sp.** | ***Radix auricularia***  **Poland** | **S** | **BU_S_162/ OM949024** | **BU_S_162/ OM949869** | **This study** |
| *Cotylurus* sp. | *Haemopis sanguisuga*  Poland | L | PG6_C_Hs/ MW204816 |  | Pyrka et al. 2021 |
| *Cotylurus marcogliesei* Locke, Van Dam, Caffara, Alves Pinto, Lopez-Hernandez & Blanar, 2018 | *Lophodytes cucullatus*  Canada | B | MH536509 |  | Locke at al. 2018 |
| ***Cotylurus* sp.** | ***Radix auricularia***  **Poland** | **S** | **BU_S_169b/ OM949025** |  | **This study** |
| *Cotylurus* sp. | *Radix auricularia*  Japan | S | LC599685 |  | Nakao and Sasaki, 2021 |
| *Cotylurus* sp. | *Radix auricularia*  Japan | S | LC599686 |  | Nakao and Sasaki, 2021 |
| *Cotylurus* sp. | *Radix auricularia*  Japan | S | LC599689 |  | Nakao and Sasaki, 2021 |
| *Cotylurus* sp. | *Radix auricularia*  Japan | S | LC599696 |  | Nakao and Sasaki, 2021 |
| *Cotylurus* sp. | *Radix auricularia*  Japan | S | LC599698 |  | Nakao and Sasaki, 2021 |
| *Cotylurus* sp. | *Radix auricularia*  Japan | S | LC599700 |  | Nakao and Sasaki, 2021 |
| *Cotylurus* sp. | *Radix auricularia*  Japan | S | LC599701 |  | Nakao and Sasaki, 2021 |
| ***Cotylurus* sp.** | ***Lymnaea stagnalis***  **Poland** | **S** | **BS_G_7a/ OM949033** |  | **This study** |
| ***Cotylurus* sp.** | ***Planorbarius corneus***  **Poland** | **S** | **ZR_S_17wra/ OM949029** |  | **This study** |
| ***Cotylurus* sp.** | ***Planorbarius corneus***  **Poland** | **S** | **ZR_S_17wrb/ OM949030** |  | **This study** |
| ***Cotylurus* sp.** | ***Planorbarius corneus***  **Poland** | **S** | **ZR_S_235/ OM949028** |  | **This study** |
| ***Cotylurus* sp.** | ***Planorbarius corneus***  **Poland** | **S** | **ZR_S_17wrc/ OM949031** |  | **This study** |
| ***Cotylurus cornutus* (Rudolphi, 1808)** | ***Anas platyrhynchos***  **Poland** | **B** | **GDK_2_6/ OM949021** |  | **This study** |
| *Cotylurus* sp | *Radix auricularia*  Japan | S | LC599706 |  | Nakao M and Sasaki M, 2021 |
| *Cotylurus* sp | *Radix auricularia*  Japan | S | LC599710 |  | Nakao M and Sasaki M, 2021 |
| ***Cotylurus* sp.** | ***Radix auricularia***  **Poland** | **S** | **BU_G_41b/ OM949038** |  | **This study** |
| ***Cotylurus* sp.** | ***Radix auricularia***  **Poland** | **S** | **BU_G_41a/ OM949037** |  | **This study** |
| ***Cotylurus* sp.** | ***Radix balthica***  **Poland** | **S** | **BJ_G_122b/ OM949039** |  | **This study** |
| ***Cotylurus* sp.** | ***Planorbis planorbis***  **Poland** | **S** | **ZP_G_136/ OM949040** |  | **This study** |
| ***Cotylurus* sp.** | ***Lymnaea stagnalis***  **Poland** | **S** | **BS_G_107a/ OM949043** |  | **This study** |
| ***Cotylurus* sp.** | ***Radix balthica***  **Poland** | **S** | **BJ_G_65/ OM949042** |  | **This study** |
| ***Cotylurus* sp.** | ***Lymnaea stagnalis***  **Poland** | **S** | **BS_G_108a/ OM949044** |  | **This study** |
| ***Cotylurus* sp.** | ***Lymnaea stagnalis***  **Poland** | **S** | **BS_G_108c/ OM949045** |  | **This study** |
| ***Cotylurus* sp.** | ***Lymnaea stagnalis***  **Poland** | **S** | **BS_S_199/ OM949047** |  | **This study** |

Table 1. The list of strigeids used in the molecular analyses. GenBank numbers of new sequences obtained in this study are in bold. Abbreviations: B - bird, F – fish, S - snail, L – leech, M – mammal

References:

Soldánová, M., Georgieva, S., Rohácova, J., Knudsen, R., Kuhn, J.A., Henriksen, E.H. et al. 2017. Molecular analyses reveal high species diversity of trematodes in a sub-Artic lake. Int. J Parasitol. 47; 327–345.

Heneberg, P., Sitko, J., Tesinsky, M., Rzad, I., Bizos, J. 2018. Central European Strigeidae Railliet, 1919 (Trematoda: Strigeidida): Molecular and comparative morphological analysis suggests the reclassification of *Parastrigea robusta* Szidat, 1928 into *Strigea* Abildgaard, 1790 Parasitol. Int. 67; 688-701.

Pyrka, E., Kanarek ,G., Zaleśny, G., Hildebrand, J. 2021. Leeches as the intermediate host for strigeid trematodes: genetic diversity and taxonomy of the genera *Australapatemon* Sudarikov, 1959 and *Cotylurus* Szidat, 1928. Parasit Vectors 14; 44.

Nakao, M., Sasaki, M. 2021. Trematode diversity in freshwater snails from a stopover point for migratory waterfowls in Hokkaido, Japan: An assessment by molecular phylogenetic and population genetic analyses. Parasitol Int. 83; 102329.

Locke, S.A., Van Dam, A., Caffara, M., Pinto, H.A., López-Hernández, D., Blanar, C.A. 2018. Validity of the Diplostomoidea and Diplostomida (Digenea, Platyhelminthes) upheld in phylogenomic analysis. Int J Parasitol. 48; 1043-1059.
